# In this issue of *Current Oncology*

**Published:** 2007-10

**Authors:** M. McLean

This issue of *Current Oncology,* the fifth of six issues scheduled for 2007, reflects the increased and continuing interest in and support for the journal. The seven original manuscripts included in the issue—reflecting a wide range of interests within the oncology community—make it one of the largest to date.

The Practice Guidelines section includes four articles: optimal chemotherapy treatment for recurrent ovarian cancer, highlighting the search for alternative agents following failure of platinum therapy; the use of Gliadel wafers in the management of malignant glioma; the treatment of depression in cancer patients, with its unique problems; and the Canadian Supportive Care Group’s consensus on the management of anemia in cancer patients. I am also delighted at the inclusion of a guest editorial, “Updates and developments in radiation oncology: times of practice change,” by Drs. Carolyn Freeman and Ervin Podgorsak. Two additional articles address the role of cancer antigen 125 in ovarian cancer surveillance and how symptom clustering is being studied in cancer patients and what the implications may be.

As announced on the journal’s Web site, www.current-oncology.com, *Current Oncology* has now been added to the list of publications accepted for Journal Selector, a database of detailed profiles of leading biomedical journals produced by PeerView Inc. that acts as a resource for authors who are deciding which journal is most appropriate for publication of their work.

